# Localisation beyond the international–local divide: paternalism and politicisation in humanitarian response in Dominica and Turkey

**DOI:** 10.1111/disa.70069

**Published:** 2026-08-16

**Authors:** Valerie de Koeijer, Bilge Sahin

**Affiliations:** ^1^ Institute of Security and Global Affairs, Leiden University The Netherlands; ^2^ International Institute of Social Studies, Erasmus University Rotterdam The Netherlands

**Keywords:** disaster, Dominica, humanitarian aid, localisation, paternalism, politicisation, refugees, Turkey

## Abstract

The humanitarian sector has been challenging power relationships between international and local actors for decades, attempting to move towards a mode of operation where local knowledge and capacity are central. The sector is thus striving to promote a more inclusive approach to aid delivery through localisation. Existing literature largely focuses on power imbalances between international and local actors. However, power imbalances among local actors can also undermine localisation efforts, meaning that affected communities do not always support or benefit from a shift in power to national and local actors. In this paper, we present a most‐different case comparison of the refugee response in Turkey and the Hurricane Maria response in Dominica, drawing on 110 interviews. We argue that dynamics of paternalism and politicisation among local actors in the same country can undermine localisation's goal of providing inclusive and needs‐based assistance. When funders do not account for such dynamics, we posit, localisation can reinforce, rather than alleviate, inequality and marginalisation.

## 1 INTRODUCTION

The international humanitarian sector is increasingly critical of the externally imposed, top‐down nature of a lot of its funding and projects (Roepstorff, 2020; Hilhorst et al., 2021; Kochanski, Scott, and Welsh, 2025). In response, it has been trying to move towards a localised approach in which local actors rather than predominantly international ones are central in providing assistance, aiming to be more inclusive and responsive to local needs. At the same time, in a striking convergence of policy directions, conservative politicians also increasingly advocate for localisation to cut aid budgets and reallocate responsibilities (Gawell, 2025; Phillips‐Barrasso, 2025).

Localisation is associated with benefits such as lowered costs of delivering aid and enhanced responsiveness to local contexts and cultures (Baguios et al., 2021; Pincock, Betts, and Easton‐Calabria, 2021). Yet, while a more localised approach to humanitarian assistance can challenge global power hierarchies reminiscent of colonial and imperial policies (see, for example, Tudor, 2023; Sondarjee, 2025), it may also reinforce inequalities within a country. Power dynamics are not confined to the international level; they continue to shape how aid policies are decided and implemented domestically. Consequently, local populations do not always welcome the transfer of aid or power to governments or other national and local institutions (Milner, Nielson, and Findley, 2016; Hirose et al., 2024).

In this paper we aim to understand why localisation does not always result in positive outcomes. Extensive scholarly discussions on localisation often focus on the relationship between local organisations and international actors (Dixon et al., 2017; Howe and Stites, 2019; Paffenholz, Poppelreuter, and Ross, 2023). In contrast, we examine power dynamics among local actors. In doing so, we contribute to the literature by highlighting power relations embedded in localisation beyond the international–local binary (Kaplan and Rhoads, 2025; Zimmermann, 2025). By keeping power central to our understanding of localisation, we argue that dynamics of paternalism and politicisation among local actors at various levels can undermine localisation's goal of providing inclusive assistance and instead exacerbate inequality and marginalisation. Comprehending these dynamics not only exposes existing power hierarchies within national and local contexts, but also reveals how they can both undermine the goals of localisation and be reinforced or even produced by localisation efforts themselves. This paper then builds on extensive work on the politicisation of aid (see, for example, Desportes, Corbet, and Siddiqi, 2025) and the limited involvement of affected communities and paternalism (see, for example, Barnett, 2016) to show how such dynamics endanger the positive effects of localisation and instead further marginalise particular groups. While the end result is that affected communities are not included, we highlight the process and different power dynamics that lead to such exclusion.

To make our argument, we present two most‐different case studies: the refugee response in Turkey; and the Hurricane Maria response in Dominica. We show that although these two cases diverge widely in terms of key characteristics such as the nature of the response, population size, and the character and positionality of the governments within international politics, the dynamics of politicisation and paternalism are strong in both instances. Our analysis draws on 110 interviews with employees of governments, international organisations (IOs), and international and local non‐governmental organisations (NGOs).

Our argument is not against localisation. On the contrary, we strongly support the transfer of power and financial resources to local actors and recognise the significant benefits this can offer. Instead, we push back against the *romanticisation* and *essentialisation* of localisation and seek to introduce greater nuance by highlighting one of its pitfalls: rather than just assuming that international donors engaging with local actors automatically makes humanitarian assistance more inclusive, localisation efforts must account for the complexities within a country or community. A truly effective approach should unpack these dynamics, ensuring that localisation does not inadvertently reinforce politicisation and paternalism among local actors. Thus, while we show that both paternalism and politicisation occur across vastly different settings, the *way* in which they manifest is context‐specific. Understanding the specificities of each context is crucial, therefore, to mitigating the issue.

The remainder of this paper begins by outlining the conceptual framework, addressing the ambiguity surrounding the definition of localisation, the question of who is considered ‘local’, and the challenges in implementing effective localisation efforts. We then present our argument and theoretical framework, illustrating how politicisation and paternalism within a country can hinder localisation. Next, we detail our case selection and methodology. This is followed by an examination of the Dominica and Turkey cases, exploring how politicisation and paternalism manifest differently in each. Finally, we present some concluding remarks.

## 2 LOCALISATION AND LOCAL ACTORS

The humanitarian sector has discussed and attempted to transfer greater power and funding to national and local actors for decades. The concept of localisation is not new; similar ideas, such as the local turn, community‐driven development, and local ownership, have long been discussed in relation to development, humanitarian assistance, and peacebuilding (see, for example, Craig and Porter, 2003; Mouelhi and Rückert, 2007; Mac Ginty and Richmond, 2013; Paffenholz, 2015; Kochanski, Scott, and Welsh, 2025). In the humanitarian field, the 2016 World Humanitarian Summit culminated in the Grand Bargain, which committed humanitarian donors and aid organisations to making aid delivery ‘as local as possible’ (Inter‐Agency Standing Committee, 2016, p. 5). Although the agreement does not explicitly define localisation, it suggests that it involves direct, quality funding for local actors, improved cost efficiency, stronger operational support, and meaningful participation of affected populations in decision making—all with the aim of expanding the reach and effectiveness of humanitarian action (Inter‐Agency Standing Committee, 2016). Localisation, in theory, operationalises the humanitarian principles by supporting local actors to shape contextually appropriate, needs‐based responses, strengthening impartiality, reducing bias, and fostering independence from external agendas (Kipfer‐Didavi, 2018).

In scholarly discussions, localisation is similarly defined as bringing local and national actors to the centre of humanitarian aid by transferring responsibilities, power, and financial resources from major international actors and funders to them, with the objective of making aid more inclusive and responsive (Bruschini‐Chaumet et al., 2019; Paffenholz, Poppelreuter, and Ross, 2023). A substantial body of literature explores what localisation entails, its rationale, and its practical implications, containing varying views on to what extent responsibilities, power, and financial resources need to be transferred to local actors. Some contributions view localisation strictly as providing direct, unconditional funding to local actors, while others also consider establishing equal partnerships between international and local organisations, or even simply hiring local staff within international agencies (Featherstone and Antequisa, 2014; Wall and Hedlund, 2016; Khoury and Scott, 2024). In this paper, we approach localisation mainly through power relations, and use Kochanski, Scott, and Welsh's (2025, p. 23) definition of the concept: ‘movements or shifts of power and agency amongst global and local actors that contribute to the transformation of local ownership and/or global norms and ideas’.

Scholars, practitioners, and policy experts have explored the positive effects of localisation, including greater contextual knowledge and cultural sensitivity, faster and cost‐effective responses, strengthened local capacity, local ownership, sustainability of assistance, improved access to hard‐to‐reach communities, and adaptability to changing contexts (Donini and Maxwell, 2013; Campbell, 2018; Konyndyk and Worden, 2019; Baguios et al., 2021). Yet, there are also persistent challenges (de Geoffroy, Grunewald, and Ní Chéilleachair, 2017; Dixon et al., 2017; Howe and Stites, 2019). The amount of funding provided to local partners directly is still limited and the 25 per cent target of the Grand Bargain has not been achieved (Metcalfe‐Hough, Fenton, and Manji, 2023). Where local partners do receive international funding, it is primarily project‐based, restricting budgetary independence (Bruschini‐Chaumet et al., 2019). Moreover, capacity‐building efforts are criticised for imposing international standards rather than integrating local expertise. Local actors also often face exclusion from decision making and are subcontracted or supervised rather than treated as equal partners (Barbelet, 2018; Fast and Bennett, 2020; Shagba, 2023; Khoury and Scott, 2024; Desrosiers and Swedlund, 2025). Consequently, much scholarship criticises the lack of achievement of localisation in practice.

Recent discussions critically examine localisation and the international–local interactions that shape who holds power, how agency is exercised, and where authority lies (Kochanski, Scott, and Welsh, 2025). These analyses show that neither the international nor the local are fixed categories; instead, power and agency move across multiple social and political spaces (Acharya, 2013). These shifts are not merely top‐down but also occur through evolving arrangements of ownership and norm transmission between actors (Kochanski, Scott, and Welsh, 2025; Zimmermann, 2025). Critical perspectives underscore that localisation can reproduce unequal power dynamics between international and local actors, particularly when pursued without acknowledging humanitarianism's colonial legacies, which influence resource allocation and beneficiary selection (Boateng, 2021). Critics further highlight the dominance of ‘outsider‐led’ approaches that fail to genuinely empower local actors, prompting calls for more ‘insider‐led’ models rooted in local ownership and leadership (Kaplan and Rhoads, 2025).

Power disparities, however, exist not only between international and local actors but also among local actors themselves (Paffenholz, Poppelreuter, and Ross, 2023). Defining who counts as ‘local’ is therefore crucial to understanding whose voices are amplified, whose needs are prioritised, and who ultimately controls resources and decision making.

Local actors include civil society groups, community‐based organisations, NGOs, municipalities, national and local governments, and entities such as faith‐based institutions, all based in the country where assistance is provided (Wall and Hedlund, 2016; Barbelet, 2018). Some also consider as local the national staff of IOs and national Red Cross and Red Crescent Societies, as well as diaspora groups, charities, and the private sector. While ‘local’ often denotes actors that are not international or foreign, it remains a highly fluid and unclear term (Ece, Murombedzi, and Ribot, 2017; Khoury and Scott, 2024). This ambiguity complicates any understanding of the ‘local’ and raises more questions about when to acknowledge affected communities as being ‘local’ (Wall and Hedlund, 2016).

The lack of a clear, consistent definition of who qualifies as local complicates power dynamics between international and local actors. Local actors are not a monolithic group but rather diverse and internally marked by their own power imbalances (Kochanski, Scott, and Welsh, 2025). They are often assumed to be embedded in the communities they serve due to their proximity and contextual knowledge (Featherstone and Antequisa, 2014; Dixon et al., 2017). Yet, as we show in this paper, we cannot assume that all national and local authorities and organisations can represent local dynamics, needs, and expectations. As Fast and Bennett (2020, p.10) state, ‘local is inherently relative – in relation to who, what or where’, and as such, ‘local may be about geography, networks, relationships or affinities, but these categories hide multiple complexities and do not provide neat distinctions’. For instance, affected communities may be distant or unfamiliar to national governments and organisations based in capital cities (Wall and Hedlund, 2016; Kraft and Smith, 2019). There are thus degrees of being ‘local’: a community‐based actor is more local than a provincial one, who is more local than a national actor. However, geographic proximity does not guarantee deep understanding of local dynamics (Robillard et al., 2020). Differences in class, ethnicity, religion, gender, and access to power and rights between ‘local’ actors and affected communities create complex dynamics, along with varied needs and expectations. Perceiving all of these actors as ‘local’ obscures these distinctions and the underlying power relations (Jayawickrama and Rehman, 2018; Robillard et al., 2020). Localisation may therefore reinforce new forms of exclusion, domination, or elite capture in the national or local context (Branch, 2014; Rhoads and Sutton, 2020; K C, 2025; Sesay, 2025). Humanitarian assistance, then, may ‘go local’ without involving the local communities and the affected people that they aim to support.

We build on this literature and show how a broad definition of who qualifies as local may enable or facilitate dynamics of paternalism and politicisation. As we show, international actors often work with more powerful actors from the country in which they implement projects. As a result, these endeavours can reinforce existing power structures through dynamics of paternalism and politicisation, all within the broadly defined category of local.

## 3 PATERNALISM AND POLITICISATION AMONG LOCAL ACTORS

Rather than focusing on the power dynamics between international and local actors in localisation (Pallister‐Wilkins, 2021), this paper draws attention to power relations among local actors themselves, particularly as they manifest through politicisation and paternalism, and explores how these dynamics can hinder the localisation process. We identify paternalist tendencies, whereby groups assume needs and interests rather than consulting with the population on such matters, and politicisation, whereby the underlying political interests of the local partner bias its decisions on the provision of assistance. We argue that both paternalism and politicisation at the local level can undermine localisation. While localisation aims to shift power from donors to recipients by empowering local actors with greater responsibility for the use and management of aid, it can also reinforce politicisation and paternalism by feeding into existing power imbalances. We focus on these two aspects specifically, because of their centrality in exposing power dynamics within a country across vastly different humanitarian settings, as supported by our case selection strategy.

Politicisation and paternalism can occur within a country even in the absence of international funding and projects. However, international funding has the potential to alleviate some of the disparities in who receives assistance nationally. In theory, international humanitarian assistance is allocated on the basis of need, without the influence of paternalism and politicisation. Yet, in a context where the government distributes assistance to a particular part of a population, international funding may still fail to reach those receiving little or no support, thereby reproducing power imbalances. Hence, while localisation seeks to shift power from international to local actors, certain local actors may retain control by continuing to marginalise vulnerable groups—either by making decisions on their behalf or by leveraging existing political, economic, and social structures to exclude them deliberately from power sharing. In the remainder of this section, we address politicisation and paternalism in turn.

First, politicisation of aid occurs when local partners pursue political objectives that undermine the equitable distribution of assistance. Although humanitarian aid is often portrayed as depoliticised, in practice, humanitarian actors navigate political challenges (Barnett, 2011; Desportes, Corbet, and Siddiqi, 2025). Such politicisation is particularly prominent in contested or violent settings (Konyndyk and Worden, 2019). Governments can favour staff and organisations based on, for instance, ethnicity, gender, religion, or political affiliation, producing disparities in aid provision for other affected communities (Donini and Maxwell, 2013). Authoritarian regimes may also invoke localisation discourse to restrict international involvement and to channel funding exclusively through regime‐affiliated organisations, consolidating their power (Robillard et al., 2020). When powerful local authorities control access to aid, international funding intended to decentralise power can instead entrench existing hierarchies. This raises questions about which local matters for humanitarian work (Fast and Bennett, 2020) and often results in elite capture involving bureaucracies, government agencies, and local power brokers (Fox, 2020).

International actors may face restrictions on the partners they can engage, particularly as host state consent is required and can be withdrawn (Sivakumaran, 2015; Swedlund, 2017). Even when such constraints are absent, chosen partners may still be politicised or have other interests. Political interest may result in the local partner steering the provision of assistance towards one part of the population. For instance, an agency may want to provide assistance only to government supporters, potentially leading to a biased aid distribution that excludes vulnerable groups based on political affiliation, thereby undermining the ultimate objective of localisation.

Second, paternalism in humanitarian aid arises when local actors assume the needs of the populations they seek to assist without consulting them or engaging local actors that are in close contact with them. We use Barnett's (2016, p. 13) definition of paternalism: ‘the attempt by one actor to substitute his judgment for another's on the ground that it is in the latter's best interest or welfare’. Affected communities face paternalistic policies in cases where decision makers in national or local organisations posit that they know better how to distribute assistance (Shagba, 2023). While the focus of paternalist tendencies in humanitarian aid is often on the international actors' behaviour towards intended beneficiaries and local actors, paternalism also can, and often does, occur among local organisations at various levels, as well as in relation to the people they intend to serve (Karaoglu et al., 2023).

Paternalism becomes especially challenging when local partners are geographically, culturally, or socially removed from the populations they seek to assist. International actors predominantly work with national‐level actors, which are far removed from affected communities themselves (Melis and Apthorpe, 2020). Disconnections between national and provincial levels are common, with the latter frequently lacking decision‐making authority (Desrosiers and Swedlund, 2025). Additionally, the local partners providing humanitarian assistance to refugees are usually NGOs that are not refugee‐led and may not share key characteristics or experiences with the people they assist (Fast and Bennett, 2020). As a result, displaced persons may not perceive these NGOs as able to address their needs adequately (Robillard et al., 2020).

Although some local organisations work diligently to understand affected communities, this is far from universal. Consequently, those receiving assistance may become further marginalised, with limited access to power and influence over aid management and delivery. Their needs often are not reflected in the support provided, while decision‐making power remains concentrated in existing hierarchies—such as urban over rural, central over peripheral, or national over refugee populations.

Paternalism and politicisation can occur at the same time, and in practice distinguishing between the two can be challenging (Barnett, 2016). Importantly, paternalist behaviour means the expectation is that actors justify what they are doing as being in the interest of the people they intend to help. This opposes purposeful steering, where local groups may advocate for a subset of the population but not the rest, on purpose. Because local partners know that international bodies want to steer clear of politicisation, they may justify their choices otherwise, making the underlying motives unclear and making it challenging to distinguish between the two dynamics at times.

## 4 CASE SELECTION AND METHODS

We present a most‐different case comparison, a case selection method in which the cases are vastly different, except for the outcome and the variable of interest (Seawright and Gerring, 2008). The two types of emergency responses examined, as noted, are the refugee response in Turkey and the Hurricane Maria response in Dominica. The authors are both experts on their respective cases.

Research in the two countries was developed separately. However, when discussing the dynamics of the operationalisation of localisation after data collection, we realised that despite the significant differences between our cases, there were striking similarities in the problems that were part of the push towards including more local actors. Consequently, we then analysed the data jointly.

The interviews in both countries included an overlap of questions that generated the data that formed the basis of this paper. These cases are most‐different, as they vary considerably across contextual factors, yet both show that paternalism and politicisation by local actors undermine the provision of inclusive, needs‐based assistance in internationally funded humanitarian responses (George and Bennett, 2005; Gerring, 2009).

Turkey is a large country in the Middle East with a population of more than 85 million and a GDP (gross domestic product) per capita of USD 15,461 (World Bank Group, 2025). We focus on its refugee response as it was hosting 3.4 million people as of December 2024, with the majority (around 3.2 million) from Syria, alongside significant numbers from Afghanistan, Iran, and Iraq (UNHCR, 2024). Dominica, in contrast, is a very small country in the Caribbean with approximately 72,500 inhabitants and a GDP per capita of USD 10,989 (World Bank Group, 2026). We focus on the response to Hurricane Maria, which devastated the island nation in 2017. The disaster claimed the lives of 65 people and caused losses and damage amounting to more than 200 per cent of the country's GDP (Government of the Commonwealth of Dominica, 2017). Moreover, while Turkey is a member of the Organisation for Economic Co‐operation and Development and the G20 (Group of Twenty) international forum, it is also becoming an increasingly important donor of international assistance (World Bank Group, 2025). Dominica, in comparison, is a very small state with little power vis‐à‐vis other nations. Given these differences, one would expect to see widely diverging dynamics in the humanitarian response. Yet, in both countries, paternalism and politicisation manifest very strongly, illustrating how such factors can threaten localisation outcomes even in vastly different contexts.

Our empirical data reveal practitioners' perspectives on localisation, centring on paternalism and politicisation, how power relations shape who is involved in decision‐making processes, how that engagement occurs, and the extent to which affected communities are genuinely considered. This paper draws on 110 interviews conducted between 2021 and 2025. In Dominica, Valerie de Koeijer held 93 interviews between 2021 and 2025 with employees of IOs and the Government of Dominica at the national and local level. In Turkey, Bilge Sahin carried out 17 interviews between 2021 and 2022 with representatives of United Nations (UN) agencies, international and Turkish NGOs, and state institutions across seven provinces.

We focused on urban and rural areas, and exclusively on internationally funded projects, in both countries. In urban areas, we interviewed staff of IOs, including UN agencies, and the national government, as these are predominantly located there. In rural areas, we concentrated on local organisations and local government actors, as they are generally included as implementing agents of internationally funded projects, and are situated in such settings. To maintain anonymity, we do not identify the specific rural areas or the names of the projects.

In Dominica and Turkey, we examined two project types: one where a UN agency implemented a project in collaboration with the government; and another where the government assumed the lead in implementing an internationally funded project. Because of the specificity of the context, in Dominica, we focused on agriculture and housing projects with a strong gender component, while in Turkey, we concentrated on support for refugee women.

We identified key organisations and government agencies operating within each country and sector and then key individuals within each organisation. We worked with a research assistant in Dominica who pinpointed key individuals and contacted them, after which we took over to schedule an interview—the option was available for everyone to decline. In Turkey, we identified the participants, scheduled the interviews, and conducted them personally.

Since we collected data (in part) during the COVID‐19 (coronavirus disease 2019) pandemic, a mixture of in‐person and online interviews took place, lasting for an average of approximately one hour. In Dominica, the interviews were conducted in English, the main working language of the country. In Turkey, they were held in Turkish; Bilge Sahin then transcribed and translated them into English. In both cases, owing to the politicised nature of the countries, we not only asked questions, but we also paid particular attention to body language and other signs of discomfort; we did not push our interviewees. For instance, in Dominica and Turkey, some interviewees laughed at certain questions rather than answering them, suggesting that they could not offer a response. Moreover, we have done our utmost to ensure the anonymity of our interviewees, except in specific cases where they explicitly stated that they wanted their position to be known.

We coded our data inductively initially, and found that paternalism and politicisation, manifesting differently across the two contexts, were a common denominator. We then linked them to existing theories and literature—most prominently, Barnett (2016) and Desportes, Corbet, and Siddiqi (2025)—before returning to the data to code deductively, to flesh out further these core concepts and perform a thematic analysis. We continued with this process iteratively, to clarify themes and produce ultimately a stable, well‐defined set. We engaged in constant comparison across cases, interview types, and research participants to avoid over‐reliance on any single perspective, and we made sure that each theme was supported by multiple data points. Our analysis of politicisation and paternalism centred around the following key issues: elite capture; centralisation of power; silencing and fear; marginalisation of local knowledge; and instrumentalisation of aid.

There is a disparity in the amount of data collected for the two case studies. For Dominica, we rely heavily on our primary data, since there is little secondary material available. People in Dominica were also open to discussing sensitive issues. In Turkey, however, it was particularly difficult to find interviewees willing to talk about such matters. Given the availability of secondary sources on political dynamics, Bilge Sahin chose to supplement the primary data from Turkey with the wealth of secondary data, thereby avoiding duplication and potential security risks to research participants.

## 5 UNRAVELLING LOCAL POWER DYNAMICS IN LOCALISATION

While there are no official definitions of what localisation entails in Turkey and Dominica, both have clear dynamics shifting power to local actors. Localisation in Turkey has unfolded through a state‐centric model that expanded after the 2016 EU (European Union)–Turkey deal. While substantial international funding was channelled into the country, humanitarian assistance remained firmly under government control, with little transfer of power or resources to local or refugee‐led organisations. Local and international NGOs were tolerated largely due to the scale of humanitarian needs and financial pressures, but their operational space narrowed as political dynamics altered (Can, 2024, pp. 24–25). This model is reinforced by the Regional Refugee and Resilience Plan (3RP), which has channelled more than USD 25 billion to the region (encompassing Egypt, Iraq, Jordan, Lebanon, and Turkey) since 2015 under Turkish government leadership. UN agencies and partners align programming with state priorities and operate mainly through government institutions, positioning the state as the primary gatekeeper of funding and participation (3RP, 2024; see also Boztaş, 2019). Funding patterns underscore this centralisation: despite humanitarian funding peaking at USD 1.28 billion in 2018, allocations to local NGOs declined sharply, and refugee‐led organisations received only 0.54 per cent of total funding (Erdilmen, 2025, p. 1548).

Owing to Dominica being less widely researched, and data lacking across the country, similar statistics are not available. Information gathered in the interviews, however, indicate that also in Dominica, the main partners of IOs are different branches within the national government. While some local NGOs have received limited funding directly, the vast majority of funds are channelled directly through the national government. In Dominica, discussions around localisation and transferring power to people within the country centre predominantly on transferring power to people in the national government; the government has also repeatedly blocked internationally funded or implemented projects until such power is ceded (as noted, for example, by an international IO employee, interview 78, 2022, and a Dominican IO employee, interview 97, 2024). As interviewees repeatedly expressed, locally operational government bodies, such as town governments, as well as local civil society organisations, were rarely included in projects implemented and/or funded by international actors.

In the remainder of this section, we discuss how paternalism and politicisation manifest in the two countries. First, we demonstrate how humanitarian assistance is politicised. In Dominica, the politicised functioning of the government has created widespread mistrust regarding the distribution of funds, including among government employees at various levels. Many interviewees perceived that aid was provided predominantly to government supporters. In Turkey, politicisation takes a more layered form. The government has created a highly controlled environment in which only government agencies and organisations closely affiliated with the state have benefited from international funding, while others have been excluded. Whereas Dominica presents a relatively straightforward case of politicised aid focused on serving citizens, the Turkish case is more complex, shaped by the presence of refugees from neighbouring countries.

Next, we focus on paternalism. In Dominica, the national government has repeatedly made decisions about post‐Hurricane Maria projects while excluding the insights of local government officers. In Turkey, by framing refugees as ‘guests’, the government has attempted to diminish the agency of that population and make refugees dependent on its ‘goodwill’. This dynamic is evident in community meetings, which are supposedly designed to hear the experiences and concerns of refugees but instead are used by state officials to instruct refugee communities on how to adapt to Turkish society. These cases illustrate how some local partners may not be aware of the issues within the community they aim to assist, and how they may have paternalistic tendencies even within the country.

### 5.1 Politicisation

#### 5.1.1 Dominica

Across the interviews in Dominica, participants (notably many from government and IOs) said that they saw assistance as heavily politicised. Dominican society is polarised along political lines, with support for either the incumbent government or the opposition often linked to family history and where a person lives. Two elements of politicisation emerged from the data: (i) the politicisation itself, and how international funds can become politicised; and (ii) the difficulty for people in Dominica to speak up about it. We address these points in turn.

Various government and IO employees explained that people who openly supported the government were much more likely to receive governmental assistance than those who backed the opposition, had openly criticised the incumbent administration, or had otherwise clashed with it. A Dominican IO employee, for instance, stated: ‘The aid, the compensation post‐Maria, was done along partisan, party politics lines. It was very clear…. If they were known to not support the government, they would either have left out completely or be given very minimal. While supporters of the government who would be very marginal farmers got tremendous amounts of compensation’ (interview 41, 2022). International IO employees expressed similar sentiments, including: ‘A challenge in Dominica is that it is very much handout‐based…. If you voted for the government, you're good. If you didn't, you're out of luck’ (interview 78, 2022). Interviewees stressed that the post‐Maria period was not an aberration, but rather representative of a longstanding practice in Dominica, extending beyond the provision of assistance. As one Dominican IO employee put it: ‘Getting certain things from the government, something you would consider routine, gets more difficult because they know who you are. If you do not support the Labour Party, you will not get through’ (interview 57, 2022).

International actors that provided assistance after Hurricane Maria operated within this polarised space. Key to their concerns is the distribution of assistance according to need, rather than as a political favour (Desportes, Corbet, and Siddiqi, 2025). Yet, interviewees repeatedly noted that they believed that if international actors gave money directly to the national government, or people or organisations closely connected to it, it would reach primarily those who supported the government. As one government officer explained: ‘Regardless of the political party in power, the IOs should not leave the distribution of these relief materials in the hands of the government…. Politicians tend to manipulate the distribution in an unjust way. The followers get the good things, and the unfollowers get the scraps’ (interview 52, 2022).

In addition, various staff working on internationally funded and/or implemented projects stated that when they tried to implement their projects, the government intervened and tried to influence who received assistance. Across projects in different sectors, interviewees described instances where the government either attempted to influence, or succeeded in influencing, who received assistance. For instance, referring to a particular project, one Dominican IO employee remarked:

*There is always the challenge of political influence. Because I had phone calls and letters written from political stance, this is the list of people I want on board, and I had to stand firm…. That parliamentary representative would like for their person to get into that project. So, there was a lot of that going on* (interview 25, 2022).

Similar statements were made about other projects and were highlighted as a major challenge for staff working on initiatives funded and/or implemented by international actors, indicating the pervasiveness of this issue.

While working with the government is important for various reasons, including respecting Dominica's sovereignty, aligning with national plans, and ensuring that projects are complementary rather than counterproductive, the politicisation of assistance was a primary concern in Dominica. Many interviewees who saw this as a major issue, however, were very careful to emphasise its sensitivity and the need to avoid their comments being traced back to them, fearing that they might face similar treatment. When asked why the distribution of aid along political lines is not openly discussed in Dominica, a former parliamentary representative of the opposition, in a high‐level position, underlined in response: ‘Because there is the fear of intimidation and victimisation. You feel that if you speak about it, you'll be intimidated and sidelined’ (interview 95, 2022).

People in Dominica were hopeful that they would receive assistance from IOs like the World Bank, which provided emergency assistance to the island nation after Hurricane Maria. Yet, where members of the population felt that funding was still controlled or manipulated by the government, or people aligned with the government, this optimism quickly faded. This occurred across multiple projects. As one Dominican IO employee noted about a particular project:

*A lot of persons applied because when they hear World Bank project, they feel it's fair. For example, if you hear UNDP [United Nations Development Programme], you feel like okay, it's going to be fair. But then when we had to explain to persons that this is just one project under the government and it's just funded by the World Bank, people talk, and people come in, and they get to realise that it's just another government project* (interview 18, 2022).

In other words, because the project was executed by the government rather than the IO, the people who did not support the government no longer felt hopeful about receiving assistance.

These examples show the highly politicised nature of assistance provision in Dominica, including attempts by the government to influence who received aid from IOs. Relying heavily on the national government, and shifting decision‐making power to it completely, would exacerbate inequalities in the receipt of assistance, and further marginalise groups that do not support the government, ultimately undermining the goals of localisation.

#### 5.1.2 Turkey

The government's approach to refugee governance in Turkey is highly politicised, often marginalising actors perceived as critical of state policies. To understand this politicisation requires tracing shifting power relations among the state, civil society, and international donors. Civil society organisations gained visibility in the 1980s and expanded after the 1999 earthquake, largely reflecting Turkey's secular state ideology (Erdilmen, 2025, p. 1548). During the 2000s, NGO proliferation accelerated, and the EU's accession process briefly widened civic space by allowing limited NGO participation in policymaking (Boztaş, 2019). This trajectory shifted with the rise of the Justice and Development Party (AKP) after 2002, as religiously motivated associations gained prominence and Islamic civil society organisations emerged as key humanitarian actors aligned with state priorities (Danış and Nazlı, 2019, p. 145). From 2015, renewed conflict, regional instability, and security concerns intensified state oversight of NGOs, leading to a centralised and tightly controlled humanitarian space. This politicised environment has deepened NGOs' dependence on international donors, as entities not aligned with government priorities are largely excluded from state‐controlled resources, forcing them to rely heavily on external support (Can, 2024, pp. 26–27).

The onset of civil war in Syria in 2011 saw many Syrians seeking refuge in neighbouring Turkey, putting pressure on its resources and their management (Ark‐Yildirim and Smyrl, 2021). In 2015, Turkey was included in the UN's response plan for Syrian refugees and signed the EU–Turkey Joint Action Plan (3RP, 2024). While funds were not directly transferred to the Turkish government, it has centralised and closely managed humanitarian aid policies and projects. Funding for refugee aid was seen by the Turkish government as an opportunity to strengthen its control over internal and foreign affairs, and localisation was intentionally constrained in favour of nationalisation, leading to exclusionary practices that marginalise both international humanitarian organisations and local stakeholders that are critical of government policies (Anholt, 2020; Brumat, Geddes, and Pettrachin, 2022; Atar, Hossain, and Ullah, 2023).

While the Presidency of Migration Management oversees refugee policies in Turkey, the Disaster and Emergency Management Presidency (Afet ve Acil Durum Yönetimi Başkanlığı, AFAD), state‐appointed provincial governors, and municipalities manage local humanitarian aid (Tan, 2022). The government restricted international NGOs and local civil society organisations from entering refugee camps and retained control over their management. The Turkish Red Crescent Society (TRCS) and the Humanitarian Relief Foundation of İnsan Hak ve Hürriyetleri (İHH) were among the few Turkish NGOs with access to Syrian refugee camps, both of which have close ties to the government (Ark‐Yildirim and Smyrl, 2021). As the number of Syrian refugees surged and many began living outside designated camps, the government strategically shifted responsibilities to organisations like AFAD, İHH, and the TRC to manage the issue and to gain access to big shares of EU and UN funding (Tan, 2022). During an interview with an employee of a civil society organisation operating under the patronage of a member of parliament (MP) of the ruling AKP, the participant repeatedly mentioned the MP's name in appreciation. She expressed praise for the Turkish government numerous times, including: ‘I appreciate them so much’; ‘I am grateful to the Turkish government’; and ‘please include my gratitude to the government in your research’ (interview 5, 2021). This participant described collaboration between her organisation and the Ministry of National Education and the Presidency of Migration Management.

In contrast, an interviewee who worked for a civil society organisation without close ties to the government reported that ‘we constantly face difficulties when trying to work with state institutions, and it is clear that the authorities are not managing humanitarian aid competently’ (interview 6, 2021). Another participant reported that ‘[w]henever we try to engage with government authorities, it's extremely difficult, our requests for meetings often go unanswered, and even when a meeting happens, nothing productive comes out of it’ (interview 8, 2022). Yet another stated that ‘the government has created its own civil society groups, and now they only invite those organisations to decision‐making meetings’ (interview 11, 2022).

Interview data show that politically curated access to humanitarian funding and decision making shapes localisation outcomes: government‐aligned organisations enjoy privileged access and influence, whereas non‐aligned NGOs face exclusion and limited engagement. This demonstrates how the state determines who qualifies as ‘local’ within the localisation agenda and who acquires resources and legitimacy.

The Turkish government, with its competitive authoritarian character, uses civil society organisations to create the appearance of civic participation in a controlled and constrained manner, ensuring that they operate within limits that support the regime's interests (Danış and Nazlı, 2019). Many of these bodies have close ties to the government, with family members of the president and other high‐level officials often holding positions on their managerial or advisory boards (Sunata and Tosun, 2019). The government uses these groups to ensure that they promote official narratives and legitimise its policies. In comparison, civil society organisations that are outside of or even opposed to having ties with the government are subjected to state repression, strict regulations, constant surveillance, and political violence (Tan, 2022). The 2016 coup attempt and the subsequent state of emergency intensified the repression, leading to increased shrinking of the civic space, the closure of more than 1,000 civil society organisations, and the imprisonment of their personnel (Yabanci, 2019). During this period, several Syrian staff members of NGOs that were shut down were deported to Syria or to third countries (Boztaş, 2019). As a result, what is framed as ‘localisation’ in Turkey functions as a mechanism for extending the government's agenda to local and refugee communities while restricting independent civil society actors and excluding refugee voices. In this context, localisation is not a shift of power downwards but a consolidation of state authority (Sunata and Tosun, 2019, p. 687).

Moreover, the political alignment of civil society organisations, whether supportive or critical of the government, influences the nature of the aid provided and their approach to refugees. According to Sunata and Tosun (2019, pp. 692–693), four types of NGOs operate within Turkey's refugee response, differentiated by scale, service models, and political ties that influence localisation outcomes. Refugee‐led and community‐based NGOs provide education, psychosocial support, medical aid, and targeted assistance to highly vulnerable groups, yet receive minimal funding owing to political marginality and limited access to resources. In contrast, large NGOs, particularly those aligned with government‐promoted religious narratives, function primarily as charitable service providers, delivering food aid, cash assistance, and basic social services in line with state priorities, and enjoying preferential access to funding. As many of these organisations are faith‐based, their orientation can exclude people of different religious backgrounds and reinforce a needs‐based rather than a rights‐focused, empowerment‐oriented model. Other independent NGOs focus on broader issues such as rights‐based advocacy for Syrians' legal protections, participation, empowerment, and efforts to break the cycle of poverty and dispossession. However, they depend heavily on international donors and face sustained political pressure. Those closest to refugee communities thus receive the least support, while larger and more politically connected actors benefit disproportionately from funding. This directly alters what kind of aid is provided and hence prevents localisation by keeping decision making away from affected communities and their actual needs.

### 5.2 Paternalism

#### 5.2.1 Dominica

In Dominica, many IOs and NGOs, including the International Organization for Migration, Samaritan's Purse, the United Nations Development Programme, and the World Food Programme, provided humanitarian assistance after Hurricane Maria. While some of these actors are often associated with development assistance, in Dominica they provided humanitarian assistance, including temporary shelter and assistance to restart food production, and all are signatories to the Grand Bargain. International actors engaged with the Government of Dominica, but often key project decisions were taken without or with very limited input from actors based within local communities, including local disaster management committees, local governments, and the affected population itself.

This subsection focuses on the national government's influence over lists of people receiving assistance, and the lack of consultation with local government actors. While these lists were politicised and often excluded people who did not back the government, paternalism still occurred regarding choices among people who supported the central administration.

Local government officers who know their communities in depth were often not consulted about who would receive assistance, or their input was disregarded. Even if the population was not consulted directly, these officers could offer detailed information. Across projects, there were instances of national‐level staff disregarding local government officers' input. At the decision‐making level of international projects, the national government was the main local actor. For example, beneficiary lists were produced in close collaboration with the national government, as an interviewee in one of the highest positions in the national government explained:

*[For one internationally funded project, my division] is very involved in terms of selection of beneficiaries. Normally what they do, they meet as a team, they go through the criteria, [my division] sometimes writes recommendations…. And that list usually comes to me, just for my information. If there is need for endorsement, I do. [For another internationally funded project,] there has to be some sort of compromise between the expectations of the [IO] in terms of beneficiary selection and the government…. And an approved list is generated. That approved list comes to me for endorsement* (interview 82, 2022).

This interviewee also highlighted that actors based within the community are consulted about who should receive assistance. However, this was not perceived as such at the local level. Instead, interviewees, including local council members and government officers based outside of the capital, repeatedly said that they were sidelined in the process. In the words of one local council representative:

*If the IOs were to go through the council to provide assistance, they would have much more of the local knowledge. For example, about people who received welfare assistance. But it didn't go through the council, and the council did not get a say…. But we really know the communities, and people lie sometimes about their situations. The council could verify this information* (Interview 35, 2022).

The in‐depth local knowledge that these employees hold, then, was not utilised.

Another local council employee raised the issue as well, highlighting that they also did not receive information about how projects were progressing. This negatively affected the local council, since it was blamed for issues but could not answer questions that emerged:

*[We] had instances where certain persons were getting houses and we were not aware. And then we are the ones being blamed. It had an instance where somebody was getting two houses. They were supposed to get one. They are getting two. And then we are the ones getting blamed, saying how come we let that pass through. And then it has other persons that need housing as well. So not being updated, it was really putting us in a bad light* (interview 102, 2024).

This exclusion meant decisions were made without the benefit of hyper‐local knowledge, while local actors bore the reputational costs. When asked why this happens, the same interviewee responded: ‘I think it's just a lack of respect for the office’.

Beneficiary selection was not the only case where local government officers were excluded from decision making. For instance, interviewees pointed out that in one internationally funded programme, many issues occurred, including the distribution of a type of fertiliser that did not work with the local soil. One local government officer explained:

*Not every [international] organisation recognises the local government officers, but they work with the national government. But the national government doesn't know the situation on the ground. And then all the decisions are taken by up there, and they don't ask about the logistics on the ground. As the [local government] officers, there are multiple issues that we could have told them* (interview 28, 2022)

While government officers operating locally have a wealth of knowledge of the type of products and approaches that would and would not work, they were not consulted, leading to issues that they say could have been avoided. Local officers highlighted that they felt people at the national level thought they knew better what decisions to make. Hence, while the focus is on the national government, they can include and consult local actors, but do not do so. Here, power dynamics between national and local government actors are once again visible, as decisions are made without the inputs of the latter.

#### 5.2.2 Turkey

In Turkey, paternalism manifests as a lack of listening combined with prescribing refugee populations how to behave. The Turkish government refers to refugees as ‘guests’ (*misafir*) to emphasise the temporary nature of their presence, framing its refugee policy around the concept of hospitality (*misafirperverlik*). This characterisation draws on religious, cultural, and national–historical narratives in the country, including Islamic traditions of hospitality and Ottoman imperial memory, to present Syrians' reception as a post‐imperial paternalist duty (Alkan, 2021). Such narratives have been leveraged by the Turkish government to position itself as a ‘caring, paternalistic state with a humane refugee policy’ (Futak‐Campbell and Küçük, 2023, p. 394). This narrative reinforces state dominance by positioning refugees as temporary visitors, dependent on the goodwill and generosity of the host nation, rather than as individuals with long‐term rights or agency (Babül, 2024). This form of framing fosters a paternalism by creating an implicit expectation that refugees have to behave well and express gratitude for the hospitality and generosity of the host country. In doing so, it positions them as passive recipients of aid, obliged to conform to the host's terms while further entrenching power imbalances. Through these paternalistic discourses in humanitarian aid, the Turkish government asserts superiority over refugees (Futak‐Campbell and Küçük, 2023). The government thus positions itself as a state that cares, and its policies are supposedly in the interest of the refugees, as they will help them to adapt to life in the country.

One forum where this paternalistic discourse was on display was in the meetings that the Turkish government organised with refugee groups. These supposedly aimed to hear refugees' experiences, but instead they often turned into an opportunity for the government to tell refugees how to behave. One such set of meetings, organised by the Presidency of Migration Management, was called Kanaat Önderleri Buluşması (Opinion Leaders Gathering). A Turkish humanitarian aid worker noted that these gatherings were presented as a chance for government officials to listen to refugees' concerns; in reality, though, they were used by state officials to communicate warnings or reminders about specific rules. The aid worker stated that in one of the gatherings he attended, ‘the mayor warned refugees not to use social media to criticise government policies, told them how they should behave on public transportation and in places like parks and markets, and even advised them to avoid eating spicy food so they wouldn't disturb their neighbours’ (interview 16, 2022). These meetings, then, reinforced power imbalances and the notion of refugees as passive recipients of humanitarian aid, in need of cultural pedagogy, and incapable of functioning as active, autonomous political agents.

Another set of gatherings also reinforced paternalism. These meetings were called Yerelde Kadın Buluşmaları (Women Gatherings in the Local) and also organised by the Presidency of Migration Management, specifically for refugee women. According to official statements, the aim of these gatherings was to promote a culture of coexistence by engaging women. However, they also become platforms for state officials to dictate what refugee women need to do and what rules they need to follow in order to live in Turkey. A humanitarian aid worker provided more details about them: ‘Participation was mandatory for the women, and the primary goal was simply to inform them, with no real intention of fostering any kind of social network among them. But they found a chance to socialise among themselves when they showcase their own local dishes to each other’ (interview 16, 2022).

While some of the information shared in these meetings was about refugee women's legal rights, the events mostly revolved around how to raise children properly. These gatherings were ‘paternalism over paternalism’ (Sözer, 2021), subjecting refugee women to a double layer of control: first as refugees being instructed on how to behave in the host country; and second as women being told how to act within the family, with officials dictating not only appropriate conduct but also their responsibilities in relation to childrearing and domestic life. As these meetings show, paternalistic care for refugee women, in turn, breeds paternalistic control—rather than shifting power from the government to affected communities by engaging in meaningful dialogue with refugee women about their struggles, needs, and initiatives for survival, refugee women were included by cooking traditional dishes and performing local dances while state authorities (predominantly men) took the lead in conversations (Presidency of Migration Management, 2019). These gendered practices remove refugee women's ability to talk meaningfully about their hardships with those providing assistance (Erman and Zadhy‐Çepoğlu, 2024) and have put them under the surveillance of the state, to ensure continually their proper vulnerable status (Sözer, 2021).

### 5.3 Comparing Dominica and Turkey

In Dominica and Turkey, politicisation and paternalism are central to understanding how power relations determine who is brought into decision‐making processes, how that involvement is structured, and the degree to which the voices of affected communities are genuinely considered. First, in both cases, the national government was the dominant local actor, concentrating power over resources and decision making. Those who challenged what they saw as politicisation were sidelined, ensuring that power remained firmly centralised. In Dominica, politicisation was rather straightforward: the government provided very little or in some cases no assistance to people who did not support it; IO staff also highlighted cases where the government tried to alter beneficiary lists for international projects. People were hesitant to speak up, as they were worried that they would be targeted, dividing society into people who did and did not support the government. In Turkey too, we see a bifurcation, predominantly among civil society organisations. They are split between government‐aligned actors that receive funding and access but reinforce government narratives that depict refugees as passive recipients of charity, and independent, rights‐based actors that hold the potential to advance localisation through inclusive and participatory practices yet are systematically excluded. In the two countries under review, those who are critical of the government or its policies are marginalised, such as through restrictive policies or, in extreme cases, imprisonment. These dynamics reveal that power relations in both settings privilege loyalty over inclusivity, directly shaping how power shifts in localisation and on what terms.

Second, paternalism further shaped the nature and quality of involvement with community‐based entities and communities themselves. In both cases, people receiving assistance and actors in daily contact with these populations, were not involved as agents. In Dominica, we identified a lack of requests for and use of feedback from local actors by the national government in the assembling of assistance recipient lists. Interviewees pointed to a lack of respect for the local councils and government officers based within communities as a reason for why they were not included; national government officers repeatedly positioned themselves as able to take decisions in the interest of the population. In Turkey, paternalism became visible in community meetings, which were highly curated spaces. These gatherings were framed as consultative, but in practice focused on the government providing information and regulating and disciplining the community, rather than listening to affected people. They illustrate how paternalism between the government and refugee population in Turkey reproduces existing power imbalances. The government then uses the language of inclusion, positing that it is in the interest of the refugee population. Yet, in Dominica and Turkey, while the national government positions itself as listening to communities and community‐based actors, in reality, its paternalist practices further marginalise refugee and hurricane‐affected populations' agency and allow the administration to stay in control.

In short, the power dynamics at play in Dominica and Turkey undermine the core intent of localisation: to shift authority and agency closer to affected communities. Instead, localisation gives more power to the national government, which suppresses critical and marginalised voices. These dynamics are not unique to these two cases, but both present a stark reminder that if deep localisation, where funds are transferred directly to local agencies, is to achieve its goal of assisting people, it is essential to comprehend how politicisation and paternalism work within a country. Consequently, providing funding requires detailed understanding of the power relations among local actors.

## 6 CONCLUSION

This paper shows that while localisation efforts are crucial for challenging global power hierarchies and making humanitarian aid more responsive and inclusive, they often overlook power dynamics among local actors and can ultimately reinforce marginalisation. While working with local actors has many benefits, this study highlights that being from the host country does not guarantee that an actor represents the needs of affected communities, as their interests and understanding of issues may differ from those receiving help.

We examined paternalism and politicisation as key factors shaping power dynamics among local actors, often undermining the goals of localisation efforts. Through case studies of emergency responses in Dominica and Turkey, two vastly different contexts in terms of geography, politics, socio‐economics, history, and humanitarian needs, we have demonstrated how these issues persist within national structures. First, paternalism is not limited to interactions between international and local actors but also exists within national and local power hierarchies. Second, political dynamics directly influence who receives aid, who distributes it, and who is excluded. Hence, this paper's contribution goes beyond the international–local binary and exposes differential power dynamics within the local.

Our argument has three main implications. First, localisation efforts, both in theory and in practice, need to include an analysis of which actors hold power within the country receiving aid. Without it, localisation risks overlooking dynamics of paternalism and politicisation, thereby undermining aid efforts. Second, international actors cannot assume that local actors, such as local councils, hold the same status or influence across contexts, making mapping of power dynamics essential before distributing funds. Third, for localisation efforts to have the desired effect and reach populations in need, projects need to account for the ways in which less powerful actors can hold more powerful actors within a country to account as key decision‐making partners.

This paper contributes to the literature on localisation, and broader humanitarianism, by pointing out the politicisation and paternalism of which international funders should be cognisant when moving to a localised approach. By distinguishing between types of local actors and examining intra‐local power dynamics, it reframes localisation as not only a shift from the international to the local, but also within the local itself. While localisation holds transformative potential for the humanitarian space, it must assess the power dynamics within countries, where paternalism and politicisation can, through the process itself, deepen rather than dismantle existing inequalities.

In light of dwindling funding, notably following the closure of the United States Agency for International Development in 2025, it is becoming increasingly important for international donors to cut costs. Providing assistance through local actors can be an important step in doing so, while also bringing myriad other benefits. The rapidly changing humanitarian field makes our argument even more pressing, as understanding dynamics of politicisation and paternalism affects who gets assistance and how. Moreover, in the face of uncertainty around sources of funding for local organisations, new actors, including private funders, are becoming more central, which can complicate power dynamics further. This paper's argument provides a starting point for analysing such changes, but more research is needed to unpack them further.

## CONFLICT OF INTEREST STATEMENT

The authors declare no conflicts of interest.

## Data Availability

Research data are not shared.
